# Medical Opinion and Movement

**Published:** 1911-03-11

**Authors:** 


### Medical Opinion and Movement.

### Treatment of Warts.

A very simple method of treating warts is advocated by Budinger, and is said to be very effective. This authority freezes the wart by spraying ethyl chloride on to it for a minute at a time, as in the familiar method of producing local anæsthesia for minor operations such as the lancing of boils, whitlows, etc. This process is repeated every other day as long as is necessary. Part of the wart thus treated drops off, and the rest gradually shrivels up, leaving a red spot. Superficial warts naturally respond to this treatment better than deeper ones; for the latter the author recommends excision of the superficial portion first, and then the ethyl chloride spraying for the deeper part. The treatment is so simple, harmless, and inexpensive, that if it fulfils all that is claimed for it a very useful weapon will be added to the general practitioner's armament.

### Asphyxia Neonatorum—Placental Aeration.

Last year Dr. Fitch reported in *Pediatrics* a new method of treatment for asphyxia neonatorum. This is a rapid delivery of the placenta, and aëration of its maternal surface. To the cases then reported Dr. Freund now adds another, the report of which is published in the *Medical Record*. His patient was a Russian primipara who was suffering from the effects of protracted labour (27 hours). In consequence she was delivered by forceps while under the influence of three doses of morphine and scopolamine, and of chloroform in addition. The child when delivered was asphyxiated, with livid skin, heart visibly beating, and cord palpably pulsating. No attempts at normal respiration took place, so the placenta was at once manually loosened and delivered. The child was placed in a bowl of warm water; the placenta was held maternal surface uppermost, washed free of blood-clot, and exposed to the air. Presently the child improved in colour, although it had not breathed at all; and the pulsation in the cord became more vigorous. After thirty-five minutes of placental aëration the surface of the placenta was placed in a current of oxygen gas, which brought about immediate recovery of pulse and colour, both of which had begun to deteriorate. The cord was then tied and cut; the child cried lustily and has behaved just like other babies ever since.

### The Tapping of Hydroceles.

At the present day there is a tendency to regard the best routine treatment of all hydroceles as that of radical cure by operation; quite lately the autoserotherapy plan has been suggested, and noted in these columns. In the *Clinical Journal* Sir Wm. H. Bennett, whose experience of these cases is enormous, lays it down as his view that about two cases out of five can be cured without operation, by simple tapping and the injection of Morton's fluid. He points out, further, that there is a form of traumatic hydrocele which supervenes within a few days of injury, such as a fall on the perineum. Such cases are distinguished from hæmatoceles by the fact that they do not come up at once, as the latter do, but only after an interval of time. Traumatic hydroceles, moreover, are less tense than hæmatoceles, and are not associated with the extensive bruising; in addition they are not opaque to light. In the ordinary way the effused fluid is gradually absorbed under rest and simple treatment. When this does not occur completely, or when there is left some induration about the epididymis, the chance of tuberculous epididymitis having supervened is one which is by no means small. It follows that great care must be exercised in allowing the patient to return to work; rest should be insisted on until the parts have quite returned to an absolutely normal condition.

### Cæsarean Section in the United Kingdom.

The whole of the January number of the *British Journal of Obstetrics* is devoted to a most valuable monograph by Dr. Amand Routh on Cæsarean section in this country, based upon the reports of 1,282 cases reported to him by the actual operators. The cases are always consecutive; that is, any operator's list is rejected unless it contains particulars of all his cases; unfortunately, several of the pioneers of this operation are now deceased, so that the table is necessarily incomplete. The cases are analysed thoroughly from every standpoint; it is only possible here to select a very few interesting items from the paper. The maternal mortality before 1891 was 35.7 per cent.; thence to 1895, 27.7 per cent.; to the end of the century, 15.3 per cent.; from 1901 to 1905 it was 13.5 per cent.; and in the past five years in over seven hundred cases it has been only 8.1 per cent. The indications held by various obstetricians to justify Cæsarean section are very numerous and diverse. It is noteworthy that contracted pelvis was the indication in 1,058 out of these 1,282 cases; fibroids account for 72 more, cancer of the cervix for 31, ovarian tumours for 28. Stenosis of the cervix led to Cæsarean section 8 times; placenta prævia 7 times; eclampsia 10 times; cancer of the rectum 8 times; pelvic tumours, such as osteomata and sarcomate, 15 times. Other indications occurred less often. For contracted pelvis the figures show plainly how important it is to operate either before labour or with the membranes unruptured. In such cases mortality (since 1891) is but 2.9 per cent.; whereas in the less favourable circumstances of ruptured membranes, frequent examinations, and attempts at delivery, the percentage of maternal deaths is 17.3.

As spokesman of British obstetric practice, the author pleads forcefully for induction of premature labour in all suitable cases. His general rule is that for true conjugates of less than 3¼ inches Cæsarean section should be urged; for those of 3¼ to 3½ labour should be induced at 35 or 36 weeks, according to the relative size of head and pelvis; and over $3\frac{1}{2}$ inches labour should be left to nature. Craniotomy is regarded with dislike except for dead children which cannot otherwise be naturally delivered and when no facilities for abdominal operations can be obtained. Maxwell's method of intra-amniotic irrigation with salt solution is highly recommended whenever craniotomy has to be performed in possibly infected patients. It should not be attempted with a true conjugate of less than 2 inches. Pelviotomy is regarded by most of the authors whom Dr. Routh circularised with great disfavour; the majority rule it out altogether, but some think it useful in certain special and occasional cases. The discussion of Cæsarean section is especially full; noteworthy also is the section on the ethics and on the desirability of sterilisation as part of this operation. As a summary of British opinion and teaching in many highly controverted questions this monograph deserves careful study from everyone who undertakes midwifery practice.

### Hair in the Treatment of Skin Wounds.

Professor Paul Carnot has for many years been a strong believer in the virtues of what he calls *hair-grafts* in the treatment of skin wounds. He returns to this subject in an article published in a recent number of *Paris Médical*. The method is based on the principle that the cells of the hair and the hair-sheaths are of epidermal origin and can easily be transformed into Malpighian cells. In addition they are hardy and used to very little nutrition, and can thus flourish and proliferate under poor conditions such as are usual when it is necessary to undertake skin-grafting. The technique of hair-grafting is simple in the extreme. A few hairs are removed with a depilating forceps from the patient's own head, or if he has none left from that of a near relative. The author states he has found that a better proliferation is secured with hairs derived from a consanguineous than from an outside source. The bulbous portion of these hairs is then cut up fine with sharp scissors, so that a kind of hair-dust is obtained, which is next put on the wound at the point where it is desired to make a graft. The wound is then covered with a sterile dressing or with Unna's paste, no strong antiseptics being used. In about a week a series of white spots appear at the place of grafting, which gradually enlarge and finally form a new skin. The author has made use of this method in his hospital practice with great success, and his article is illustrated with cases in which he has, by its use, brought about a speedy cure in very severe cases of loss of skin from burns, varicose ulcers, etc.

### Reform of Medical Education in France.

In view of an article on this subject that recently appeared in this Journal, it is interesting to note that the following resolution was recently passed at a general meeting in Paris of the Co-Operative Association of Medical Students: That this meeting, approving the attitude of the committee, in particular as regards questions of medical education, is determined to pursue energetically the fight for the improvement in professional instruction for students (an improvement the necessity of which is admitted by the whole of the body medical), regrets that no reform has as yet been instituted by the public authorities, and demands: (1) the limitation of the number of students in each clinic to twelve, a limitation which will necessitate an increase in the number of clinics, and (2) the creation of a superior medical council composed as to one half of professors of the faculty, and as to the other of delegates of the medical syndicates, with some means of consultation for the corporate bodies of medical students. But considering that the organisation of professional teaching necessitates a more thorough reform of the hospital stage, the meeting demands that an enquiry be held with a view to effecting modifications in the office of externe, which would then become an appointment open to all students after examination. All medical students would be obliged to have held this appointment for at least two years before obtaining the degree of Doctor in Medicine.

### Subcutaneous Injections of Oxygen.

Reference has already been made in these columns to the administration of oxygen by subcutaneous injection in conditions of asphyxia such as would in the ordinary way be treated by oxygen inhalations. Further reports are now to hand which appear to support the claims of efficacy previously made for the treatment. Dr. Maisonnet, of the military hospital of Val-de-Grâce, has tried the method in five cases, including post-operative conditions after operations, pneumonia, and bilateral purulent pleurisy. The following is the method adopted: The skin on the external surface of the thigh is sterilised with tincture of iodine and a sterilised injection-needle introduced into the subcutaneous tissue, care being taken to ascertain that it has not penetrated a vein. To this is fitted an indiarubber tube connected with a bag containing the oxygen and fitted with a glass tube containing sterilised cottonwool to filter the gas. By compressing the bag the gas passes slowly into the subcutaneous tissue. The duration of the injection is about twenty minutes. The volume has not been measured, but the flow of gas may be continued till a swelling is formed similar to that caused by an injection of 300 grammes of serum. After the injection gentle massage should be applied to cause diffusion and absorption of the gas. The injections may be repeated twice in the same day. As a result, the dyspnœa and cyanosis rapidly disappear and the patient has a feeling of well-being. One patient expressed himself as feeling "refreshed inside." The respirations diminish in rapidity and their amplitude increases. The pulse-rate also is usually diminished and the general condition improves, so that the oxygen seems to act as an antitoxic agent as well as an oxidiser.

Dr. Sacquepee, Professor of the Military Medical School of Paris, also reports favourably on the treatment. All the cases were infections with the pneumococcus. In all these cases the injections of oxygen were followed by marked improvement in the patient's condition, which generally continued for about twelve hours after the injection. The circulation and respiration were especially favourably affected. In the course of three to five hours afterwards the pulse-rate fell by ten to twenty pulsations a minute, and the blood pressure was also diminished. The respiratory rate fell often by as much as a third or a quarter of the number of respirations per minute. The injections have no influence upon the temperature or the pulmonary lesions and course of the disease, but by repeating them the patient is kept in a much better general condition and is presumably better able to withstand the severity of the disease. At the time of the crisis the author is of opinion that the injections are especially efficacious in attenuating the severity of the condition and preventing the occurrence of syncope or other dangerous symptoms.

### Paralysis Agitans.

Recent researches and observations point to a possible connection between the parathyroid glands and paralysis agitans. Symptoms observed as a result of parathyroidectomy are very similar to those of Parkinson's disease, and the disease may occur as a complication or sequela in cases of myxœdema or exophthalmic goitre. Finally, degenerative lesions of the parathyroid glands have been observed in cases of paralysis agitans. In view of these facts, Dr. Berkeley, of New York, has tried opotherapy with parathyroid glands in cases of paralysis agitans. Altogether he has treated sixty cases of the disease. In about 65 per cent. of the cases in which he has been able to continue the treatment for a sufficiently long time, he has obtained marked improvement. In more than a dozen patients who have had the treatment for three or four years this improvement has been so definite that the symptoms are no longer apparent except when the treatment is interrupted. Some of them appear to be almost completely cured, so far as one can speak of a "cure" in cases of this disease. At first Dr. Berkeley employed fresh glands triturated with an excess of lactose and then put up in capsules, but he found that they were difficult to preserve. Since then he has used an extract of the nucleo-proteids of parathyroid glands, obtained by the method of Beebe. This product is in the form of a yellow powder, which may be mixed with lactose and put up in capsules, each containing 0.0012 gramme of parathyroid nucleo-proteid. The dose consists of one or two capsules a day. Sometimes the treatment produces a certain nervous excitability and increases the habitual constipation of these patients. In such cases the dose must be diminished and then gradually increased again.

### Urethral Stricture.

Dr. Frank's method of treating post-operative paralysis of the bladder with retention of urine by means of glycerine injection has been recently described in these columns. Dr. Goldenberg, of Nuremberg, has employed a similar method to overcome "impassable" strictures of the urethra. He first used it two and a half years ago in cases of urethral stricture which did not yield to ordinary methods of procedure. A filiform catheter was passed as far as the stricture and then a large ordinary glass syringe was fitted to the urinary meatus and given to an assistant, who compressed the walls of the meatus against the orifice of the syringe with one hand and with the other hand worked the piston. At the same moment that the assistant forced in this way about 15 cubic centimetres of glycerine into the meatus, Dr. Goldenberg pushed forward the catheter, which then easily overcame the stricture and entered the bladder. The author has since used the same method successfully in several cases of stricture and of obstruction from prostatic hypertrophy. The author points out that the method is simple and innocuous. Sometimes hæmorrhage results from slight rupture of the cicatricial tissue of the stricture. The glycerine has the advantage of being soluble in water, and consequently there is no danger of embolism as in the case of oily substances. Any acute inflammation is, of course, a contra-indication to the use of the method.

### Dental Caries in Children.

In an article on the treatment of dental disease in children published in the *Dental Record*, Dr. J. F. Colyer has some distinctly practical suggestions to make on the question of the prevention of dental caries. To some extent he agrees with Dr. Wallace in thinking that the ridding of foodstuffs of their fibrous parts by modern methods of preparation renders them more liable to lodge about the teeth and so ferment, with consequent tendency to bring about caries. But there is another aspect of the food question which must not be lost sight of, viz. the remarkable change of late years in the character of the carbohydrates, e.g. sugar and flour, which undergo fermentation much more readily than formerly. He unreservedly condemns biscuits for young children, though he considers sugars and the mono-saccharides in particular even more harmful than these. Preventive treatment should follow the following lines: (*a*) the insistence on breast-feeding where possible; (*b*) the use from early years of foodstuffs requiring efficient mastication; (*c*) the use of carbohydrates that are not easily fermentable, and the forbidding of sweets in the form of sweetmeats. The use of the tooth-brush, while of secondary importance to the above, should be dogmatically insisted upon. Care should be taken that the mouth is functional, for which two things are necessary—namely, absence of mouth-breathing and of tender teeth. The former causes a persistent marginal gingivitis around the incisor teeth, and the latter lead to oral sepsis. If the pulp is exposed, he advises extraction, and only uses conservative treatment in cases where caries has not proceeded to this extent.

